# Recurrent erysipelas - risk factors and clinical presentation

**DOI:** 10.1186/1471-2334-14-270

**Published:** 2014-05-18

**Authors:** Malin Inghammar, Magnus Rasmussen, Adam Linder

**Affiliations:** 1 Department of Clinical Sciences, Division of Infection Medicine, Klinikgatan 1, Skåne University Hospital, Lund, Lund SE-221 85, Sweden; 2 The Division of Health Surveillance and Research, Statens Serum Institut, 5 Artillerivej, Copenhagen DK-2300, Denmark; 3 Division of Critical Care Medicine, Centre for Heart Lung Innovation, St. Paul’s Hospital, University of British Columbia, 1081 Burrard Street, Vancouver, BC V6Z 1Y6, Canada

**Keywords:** Erysipelas, Skin infection, Recurrent, Risk factor

## Abstract

**Background:**

Erysipelas is a common infection that often recurs, but the impact of specific risk factors for reoccurrence remains elusive. In the present study we aimed at clarifying predisposing conditions for reoccurrence.

**Methods:**

Medical records were reviewed from all patients ≥18 years of age diagnosed with erysipelas at the Department of Infectious Diseases at Skåne University Hospital, Sweden, from January 2007 to February 2011. 502 patients were included, of which 357 were single episode erysipelas and 145 had recurrent erysipelas. These two groups were compared regarding underlying conditions and clinical presentation.

**Results:**

Erysipelas in the lower limbs had the greatest propensity of recurrence. The associations between underlying conditions and recurrence were largely depending on the site of erysipelas. Overall, the most prominent risk factor for recurrence was lymphedema and other conditions causing a chronic impairment of the defence against microbes. Conditions temporarily disrupting the skin barrier (*e.g*. a local wound or toe web intertrigo), although likely being risk factors for erysipelas *per se*, did not seem to predispose to repeated episodes. Individuals with recurrent erysipelas tended to seek medical attention earlier, and were less likely to be hospitalized or receive intravenous antibiotics, but there was no evidence of any difference in inflammatory reaction when taking confounding factors into account.

**Conclusions:**

In this large cross-sectional study of over 500 patients with erysipelas, lymphedema was the most prominent risk factors for recurrence although the distribution of predisposing conditions varies depending on the site of erysipelas.

## Background

Erysipelas is a common infection of the superficial layer of the skin, in contrast to cellulitis and necrotizing fasciitis, which also involve the subcutaneous tissue. It has an estimated incidence of 19–24 per 10,000 inhabitants in European countries [1,2]. Erysipelas is defined as an acute onset of local signs of inflammation such as progressing erythema, associated with pain and swelling, clearly demarcated from the surrounding tissue. In the typical case erysipelas also manifests with systemic symptoms such as fever, chills and malaise and sometimes accompanied by nausea and vomiting [3,4]. The main causative agent is group A and group G β-hemolytic streptococci. Whether erysipelas also can be caused by *Staphylococcus aureus* or gram-negative bacteria is debated [5-7]. The most common site of the infection is the lower limb, accounting for about 80% of all cases [8]. In general erysipelas is a mild disease with low case-fatality, but a significant part of patients need in-hospital treatment [9]. The most common complication is recurrence, which occurs in 12-29% of the cases [5,10,11].

There are various risk factors reported for erysipelas including disruption of the cutaneous barrier, venous insufficiency, lymphedema and overweight [12,13]. In the upper limbs, lymphedema or radical mastectomy are important risk factors [14], whereas risk factors for recurrence are less well defined [15]. Prophylaxis with penicillin V is sometimes recommended, but treatment criteria are not well defined. This common infection causes both suffering and medical expenses that should motivate appropriate prevention, which should be directed to patients with the greatest risk [1]. In this study, we hypothesized that patients with single episode erysipelas and patients with recurrent erysipelas differs regarding general characteristics and we aimed to clarify risk factors for erysipelas recurrence. We also aimed at assessing whether the clinical presentation in episodes of recurrent erysipelas differ from single episodes.

## Methods

We identified all patients ≥ 18 years of age, both hospitalized and outpatients, diagnosed with erysipelas at the Department of Infectious Diseases, Skåne University Hospital, Lund Sweden, between January 2007 and February 2011. The hospital is a university hospital serving 200,000 inhabitants, living in the city of Lund (southern Sweden) and surroundings. The diagnosis of erysipelas was determined by the admitting physician. The medical records were reviewed and data extracted according to a pre-specified protocol, all data was anonymized before analysis.

The patients were divided into two groups for comparison, single episode erysipelas (SE) and recurrent erysipelas (RE). SE was defined as patients with erysipelas without a history of previous episodes (medical records and anamnestic information). RE was defined as patients with more than one episode of erysipelas registered during the study period, or anamnestic information of previous episode outside the study period. Parameters are shown in the Additional file 1. Malignancy included both prior and present occurrence of all types. Previous regional operation was defined as surgical interventions at the area affected by erysipelas or adjacent locations. As a number of patients from the RE group was registered for more than one episode (separated by at least 30 days) during the study period, the medical record from the last recurrent episode that occurred within the study period was selected for comparison with the SE group.

The Ethics Committee of Lund University, Sweden (Dnr. 2011/674) approved of the study.

### Statistics

Fisher’s exact tests, Chi-squared tests and analysis of variance were used to assess the distribution of background factors in individuals with SE and RE, and to assess the distribution of markers of disease severity and clinical presentation between individuals with SE and RE. Multiple imputation was used to handle missing data, using chained equations with 20 imputation sets, see appendix for details [16,17].

Logistic regression and exact logistic regression was used to assess predictors of recurrence. We considered gender, age (linear), localization (lower limbs, upper limbs, thorax/trunk, head/face) local operation (any kind), radiation therapy, severe obesity, liver disease, autoimmune/systemic inflammatory disease, immuno-suppressive therapy, peripheral arterial insufficiency, peripheral venous insufficiency, lymphedema, wound, toe web intertrigo, diabetes mellitus, chronic obstructive pulmonary disease (COPD), cardiovascular disease, and polyneuropathy. Covariates assessed in univariable models were considered for inclusion in multivariable models at a p-value of <0.2. Due to the anticipated modifying effect of erysipelas site, separate models were fitted for lower limbs, upper limbs, thorax and trunk, and head or face. Akaike’s information criterion was used to assess model fits and Hosmer and Lemeshow’s goodness-of-fit test in the complete case analyses was used to test the final models.

Logistic regression was used to assess whether individuals with recurrent episodes had a milder or more severe disease presentation, as measured by the need for hospitalization and/or initial intravenous antibiotics. Age, localization, time since onset of symptoms (<12 hours, 13–72, >72) were included. Linear regression, adjusted for site and time since onset, was used to assess differences in C-reactive protein levels (CRP), (square root of CRP) and white blood cell counts (WBC), (log value of WBC). Likelihood ratio tests were used to test for differences and interactions. All analysis was performed with STATA/SE (version 12.1; StataCorp LP, USA).

## Results

### Patient demographics

During the study period, 601 patients were registered with the diagnosis of erysipelas. Among them, 99 were excluded due to medical records that could not be retrieved, initial erysipelas diagnose that changed to other diagnose during the episode and non-acute patient visits.

A total of 573 episodes of erysipelas in 502 patients were identified during 2007–2012. The median age was 60 years (range 18–97), 58% were male. The infection was located in the lower limbs in 69% of the episodes, in the upper limbs in 14%, on the thorax or trunk in 8% and in the head or face in 9%. The episodes were evenly distributed over the year, with no accumulation in either cold or warm months. Overall 64% received initial intravenous antibiotic treatment, and 55% were hospitalized; 30% had a positive local culture and 5% were bacteraemic.

Three hundred sixty-nine individuals had no previous history of erysipelas and 133 had a positive history. Forty-six individuals experienced at least one recurrence during the study period (median 3 episodes, range 2–5). Nine of these had no previous history but experienced recurrence during the study period.

### Clinical presentation and disease severity

Individuals with SE, n = 360 (71%), were compared with those with RE, n = 142 (29%). Neither age (p = 0.06) nor the sex distribution (p = 0.36) differed between the groups. Individuals with RE were more prone to seek medical care earlier, 33% <12 hours since onset of symptoms vs. 17% in individuals with SE (p = 0.01), and were less likely to be hospitalized, adjusted odds ratio (aOR): 0.5 (95% CI: 0.3-0.8) or receive initial intravenous antibiotic treatment aOR: 0.6 (95% CI: 0.4-0.9), estimated by logistic regression adjusted for age, localization, time since onset of symptoms and underlying co-morbidity. However, there was no difference in the distribution of the level of CRP (p = 0.90) or white blood cell (WBC) counts (p = 0.58), estimated in linear regression models adjusted for time since onset and localization. In addition, there was no difference in the length of antibiotic treatment (p = 0.78) nor in the length of stay (LOS) – analysed among those hospitalized (n = 278), aOR for LOS >7 days for RE was 1.1 (95% CI 0.5-2.3) adjusted for age, severe obesity, malignancy, COPD, diabetes and localization (p = 0.93). See Table 1 for crude estimates.

**Table 1:** Clinical presentation in individuals with first and recurrent episodes of erysipelas

	**Single episode**	**Recurrent episodes**	**p-value**
	**n = 360 (100%)**	**n = 142 (100%)**	
Age, years (SD)	58.0 (18.7)	61.5 (16.1)	0.05
Gender,% male	211 (58.6)	75 (52.8)	0.24
*Time since onset*			<0.01
<12 hrs	47 (13.1)	39 (27.5)	
13-48 hrs	144 (40.0)	49 (34.5)	
>48 hrs	85 (23.6)	29 (20.4)	
Missing	84 (23.3)	25 (17.6)	
*Localization*^*1*)^			<0.01
Lower limbs	231 (64.2)	111 (78.2)	
Upper limbs	60 (16.7)	15 (10.6)	
Thorax/trunk	24 (6.7)	11 (7.6)	
Head/face	45 (12.5)	6 (3.5)	
CRP, median (range)	88 (0–513)	66 (2–438)	0.28
Missing	57 (15.8)	29 (20.4)	0.22
WBC, median (range)	11 (2.7-68)	13 (2.6-28)	0.82
Missing	73 (20.3)	31 (21.8)	0.70
Inpatient treatment	211 (58.6)	67 (47.2)	0.03
Initial iv antibiotics	239 (67.0)	84 (59.6)	0.18
Length of hospital stay, median (range)	5 (1–34)	5 (1–22)	0.61

^1)^-Global p-value from likelihood ratio test <0.01).

### Risk factors for recurrent erysipelas

The localization of erysipelas was associated with RE in univariable analyses, as were venous insufficiency, lymphedema, severe obesity, previous operation and a history of malignancy, whereas a local wound and COPD was positively associated with SE, see Table 2. As anticipated, there was a strong heterogeneity (*i.e.* effect modification) caused by the site of erysipelas.

**Table 2:** Distribution of underlying factors in individuals with single or recurrent episodes of erysipelas

	**Single episode**	**Recurrent episodes**	**Odds ratio (95% CI)**	**p-value**
	**n = 360 (100%)**	**n = 142 (100%)**		
Age, years (95% CI)	58.0 (56.1-60.0)	61.5 (58.6-64.1)	1.1 (1.0-1.2)	0.05
Gender, % male	211 (58.6)	75 (52.8)	0.8 (0.5-1.2)	0.36
Local operation	71 (19.7)	46 (32.4)	2.0 (1.3-3.0)	<0.01
CABG^1)^	16 (4.4)	13 (9.2)	2.2 (1.0-4.6)	0.05
*Localization*				
Lower limbs	231 (64.2)	111 (78.2)	1 (reference)	<0.01
Upper limbs	60 (16.7)	15 (10.6)	0.5 (0.3-0.9)	
Thorax/trunc	24 (6.7)	11 (7.6)	0.9 (0.4-2.0)	
Head/face	45 (12.5)	6 (3.5)	0.3 (0.1-0.7)	
Skin disease	42 (11.7)	24 (16.9)	1.5 (0.9-2.6)	0.12
Malignancy	53 (14.7)	36 (25.4)	2.0 (1.2-3.7)	<0.01
Radiation therapy	17 (4.7)	11 (7.8)	1.7 (0.8-3.7)	0.19
Immuno-suppressive therapy	36 (14.1)	8 (8.2)	0.5 (0.2-1.2)	0.14
Arterial insufficiency	13 (3.6)	8 (5.6)	1.6 (0.6-3.9)	0.32
Venous insufficiency^1)^	16 (4.4)	16 (11.3)	2.7 (1.3-5.6)	<0.01
Lymphedema	8 (2.2)	19 (13.4)	6.8 (2.9-15.9)	<0.01
Diabetes mellitus	52 (14.4)	25 (17.6)	1.3 (0.8-2.1)	0.38
Polyneuropathy	11 (3.1)	4 (2.8)	0.9 (0.3-2.9)	0.89
Vascular disease	62 (17.2)	24 (16.9)	1.0 (0.6-1.6)	0.93
COPD	22 (6.1)	3 (2.1)	0.3 (0.1-1.1)	0.09
Systemic inflammatory disease	16 (4.5)	4 (2.8)	0.6 (0.2-1.8)	0.41
Wound	176 (49.0)	53 (37.3)	0.6 (0.4-0.9)	0.02
Toe web intertrigo^1)^	10 (2.8)	8 (5.6)	2.1 (0.8-5.4).	0.13
Severe obesitas	1 (0.3)	4 (2.8)	10.4 (1.2-93.9)	0.04
Liver disease	9 (2.5)	4 (2.8)	1.1 (0.3-3.7)	0.84

^1)^-Calculated in the 342 individuals with erysipelas in the lower limbs, 229 and 113 respectively.

In univariable analyses of erysipelas in the lower limbs (n = 342) local factors, i.e. skin disease, venous insufficiency and lymphedema were predictive, see Table 3. In multivariable analyses, there was statistical evidence for the association between RE and lymphedema, aOR 4.3 (95% CI 1.3-14.0), venous insufficiency aOR 2.3 (95% CI 1.0-5.2), skin disease aOR 1.9 (95% CI 1.0-3.7) and COPD, OR 0.2 (95% CI 0.04-1.0). However, effect estimates indicated a four-fold increased risk of recurrence for severe obesity and a two-fold for previous CABG, although these did not reach statistical significance.

**Table 3:** Comparison between predisposing conditions of erysipelas in the lower limbs

	**Single episode**	**Recurrent episode**	**OR (95% CI) from univariable analysis**	**p-value**	**OR (95% CI) from multivariable analysis**^**1)**^	**p-value**
	**n = 231 (100%)**	**n = 111 (100%)**	**(95% CI)**		**(95% CI)**	
CABG	11 (4.8)	11 (9.7)	2.2 (0.9-5.2)	0.08	2.0 (0.8-5.1)	0.12
Skin disease	26 (11.3)	23 (20.7)	2.1 (1.1-3.8)	0.02	1.9 (1.0-3.7)	0.05
Severe obesity	1 (0.4)	4 (3.6)	8.5 (0.9-77.9)	0.06	4.6 (0.5-45.8)	0.19
Venous insufficiency	14 (6.1)	16 (14.4)	2.6 (1.2-5.6)	0.02	2.3 (1.0-5.2)	0.05
Lymphedema	6 (2.6)	9 (8.1)	3.3 (1.1-9.5)	0.03	4.3 (1.3-14.0)	0.02
Local wound	118 (51.1)	49 (44.1)	0.8 (0.5-1.2)	0.23	0.8 (0.5-1.3)	0.42
COPD	15 (6.5)	2 (1.8)	0.3 (0.06-1.2)	0.08	0.2 (0.04-1.0)	0.05
Diabetes mellitus	44 (19.1)	20 (18.0)	0.9 (0.5-1.7)	0.82		
Malignancy	20 (8.7)	14 (12.6)	1.5 (0.7-3.1)	0.29		
Toe web intertrigo	10 (4.3)	8 (7.2)	1.7 (0.7-4.5)	0.27		
Local operation^2)^	49 (21.2)	27 (24.3)	1.2 (0.7-2.0)	0.28		

^1)^-Adjusted for all of the variables in the column; ^2)-^CABG included.

In erysipelas located in the upper limbs (n = 75), gender, malignancy, local operation, lymphedema and local wound were predictive in univariable analyses. Due to the complex relationship between malignancy, operation and lymphedema, multivariable analyses were performed in three steps. In the first model, gender, malignancy and wound was included, in the second, operation and radiation was added, and thirdly lymphedema. The effect of gender in univariable analysis was completely confounded by a history of local operation. The final model included only operation and lymphedema. All of the effect of malignancy on the propensity for recurrence was mediated by a history local operation, aOR: 13.2 (95% CI 2.0-85.4) which in turn was partly mediated by the presence of lymphedema, aOR: 7.4 (05% CI 0.8-66.1). In univariable analyses, the OR for lymphedema (all patients with lymphedema had been operated on) was 43.5 (95% CI: 7.5-249.7). Although analyses were based on small number, the OR for local operation, from stratified analysis in individuals without lymphedema, was 28.0 (95% CI 3.3-236.4), which supports the idea that a local operation is a strong risk factor for recurrence and that the effect is enhanced by the development of lymphedema.

When erysipelas was located on thorax or the trunk (n = 35), only malignancy, OR 10.0 (95% CI 1.1-90.8), and wound, OR 0.09 (0.0-0.6), were associated with RE.

In erysipelas located in the head or face (n = 50), only 14% were RE and none of the examined covariates were associated with recurrence in univariable analyses, data not shown.

### Complete case analyses

Missingness for CRP, WBC and time since onset of symptoms was highly dependent on hospitalization, but evenly distributed among individuals with RE and SE. The distributions of values from complete cases and imputed values in different strata of hospitalization were very similar. Estimates from additional analyses using only complete cases yielded very little deviation from analyses including imputed values and did not change the conclusions drawn, see Additional file 1.

## Discussion

In this large study of over 500 patients with erysipelas, we found lymphedema to be the most prominent risk factors for recurrence although the distribution of predisposing conditions varies depending on the site of erysipelas. In erysipelas in the head or face, none of the investigated factors were associated with recurrence.

There are only a few studies regarding risk factors for RE, although previous results are generally in line with ours, most previous studies have neither been powered enough to allow for multivariable adjustments for coexisting risk factors nor to assess effect modification by the site of erysipelas [11,15,18-21]. Predisposing conditions for recurrent erysipelas located in the head or face has to the best of our knowledge not been studied previously. Our results show that there is a strong heterogeneity in the estimates from different sites of erysipelas, both in the propensity for recurrence and among the individual risk factors for recurrence, therefore analyses must be done separately for each site.

**Figure 1:**
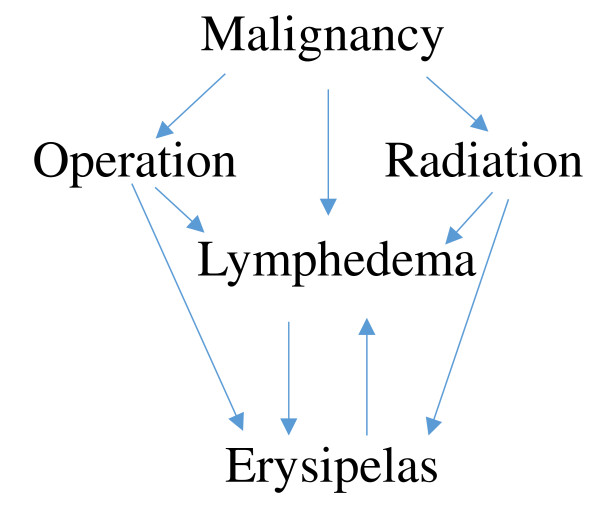
Directed acyclic graph (DAG) over relationship between predisposing conditions in erysipelas of the upper limbs.

It is well known that women treated for breast cancer are at risk for ipsilateral erysipelas, especially if lymphedema develops [14,22]. Pavlotsky *et al.* found cardiac disease, malignancy and lymphedema to be associated with RE in the upper limbs in univariable analyses, but effect sizes are not given [18]. We found local operation and lymphedema to be strongly associated with RE, although it is difficult to obtain reliable estimates due to the complex relationship between tumor, surgery performed, radiation therapy, lymphedema and infection as outlined in Figure 1. In line with previous findings, we did not find evidence for radiation therapy to be a risk factor for recurrence [23].

Oddly, we found COPD to be protective against RE in the lower limbs even when adjusting for multiple covariates, this might be a spurious association or, more speculatively, due to a high use of antibiotics directed against airway pathogens in these patients which could reduce the colonizing burden of causative agents, *e. g.* group A streptococci.

It has been suggested that RE has a more severe disease presentation as measured by peak CRP and WBCs, length of fever and length of hospital stay [15]. However, these estimates were not adjusted for time since onset of symptoms or site of infection. We did not find evidence for differences in inflammatory parameters when taking time since onset of symptoms and potential confounders into account. Rerunning analyses, including only inpatient treated individuals, yielded similar results. It is possible that the lower propensity for hospitalization in patients with RE could be attributed to a higher threshold among doctors for hospitalization and treatment with intravenous antibiotics, since patients with RE have experience of the disease.

This study has several strengths. It is one of the largest of its kind, which permits analyses of erysipelas per site of infection as well as adjustments for coexisting risk factors. We based our analyses on data collected from the medical charts, thus there was no risk of biased recall of information. Swedish health care is publicly financed and all inpatient care is provided independently of health insurance and the patient’s financial status. A unique, lifelong ten-digit personal identity number assigned to each person living in Sweden provides the possibility of linking records in health care databases which limits the risk of biased selection. Erysipelas is by tradition cared for, at Skåne University Hospital, by specialists of infectious disease. We included all patients who were diagnosed with erysipelas at the Department of Infectious Diseases at Skåne University Hospital, during the study period.

There are also some limitations to this study. The retrospective design allow only available information restricted to medical records. Data were extracted according to a pre-specified protocol but some covariates may still be subject to interpersonal differences in the assessments.

The fraction of missing information on BMI was 81%, why it was not considered reliable to impute values. Instead information on “severe obesity” was used, which was considered highly specific but with low sensitivity. If BMI-values for all participants had been available, the point estimates for obesity (as defined by BMI-values), would probably have been statistically significant but diluted.

Patients were admitted to the Department of Infectious Diseases, although both as outpatient and inpatient, there is likely to be a bias towards patients with more severe disease. If the propensity to be admitted at the department of infectious disease is differential for patients with SE and RE, estimates could have been biased.

During the average two-year period of follow up, 3.3% of the primarily naïve patients, experienced recurrence. Some of the patients classified as SE will probably later experience recurrence, this misclassification will bias estimates towards the null.

## Conclusion

This study shows that a large proportion of cases with erysipelas are RE but these episodes do not seem to be more severe than SE. The most prominent risk factor for recurrence is lymphedema, regardless of the site of erysipelas, and other conditions causing a chronic impairment of the defence against microbes. Whereas conditions, temporarily disrupting the skin barrier (*e.g*. a local wound or toe web intertrigo), although being risk factors for erysipelas *per se*, did not seem to predispose to repeated episodes.

## Competing interests

The authors declare that they have no competing interests.

## Authors’ contributions

Conception and design: MR, AL, Acquisition of data: EH, EK MR. Analysis and interpretation of data: MI, MR, AL. Drafting or revising the article: MI, MR, AL. Final approval of the manuscript: MI, MR, AL.

## Pre-publication history

The pre-publication history for this paper can be accessed here:

http://www.biomedcentral.com/1471-2334/14/270/prepub

## Supplementary Material

Additional file 1 Appendix. Click here for file
