# Supplementary material for: Recurrent erysipelas - risk factors and clinical presentation
Source: BMC Infect Dis. 2014 May 18;14:270. doi: 10.1186/1471-2334-14-270 (PMC4033615; doi:10.1186/1471-2334-14-270)
Supplement: Additional file 1 — Appendix. [file 1471-2334-14-270-S1.docx]

**Appendix**

**The following parameters were registered for all patients:**

Date, recurrence, age, sex, height, weight, outpatient/hospitalized, number of visits if outpatient, length of hospitalization, time from onset of symptoms to treatment, affected anatomical region and symptoms.

Initial values for: temperature, heart rate, blood pressure, respiratory rate and initial laboratory analysis: Haemoglobin count, WBC, CRP, platelet count and blood creatinine level.

Anticoagulant treatment, immune-supressive treatment, dermatologic diseases, toe web intertrigo, occurrence of wounds, previous operations and types, CABG, previous malignancies and types, radiation therapy, arterial insufficiency, venous insufficiency, lymph oedema, diabetes, polyneuropathy, cardiovascular diseases, COPD, systemic diseases, penicillin drug reaction, antibiotic treatment, other medical treatment, blood culture, wound culture and complications.

**Multiple imputation:**

The pattern of missing values was carefully analysed, and missing values were assumed to be missing at random. Sensitivity analyses were performed examining the effect of different assumptions about the underlying mechanism behind the missing values [[16](#_ENREF_16)]. All variables in the main analysis and all variables predictive of and influencing the missing values were included in the imputation model (CRP, WBC, arterial insufficiency, skin disease, COPD, local wound, intravenous antibiotics, time since onset of symptoms, RE/SE, age, gender, hospitalization, malignancy, radiation, lymphedema, venous insufficiency, local operation, diabetes mellitus, vascular disease, inflammatory disease, severe obesitas, liver disease, toe web intertrigo) [[24](#_ENREF_24)]. All analyses were performed using STATA 12.0, College Station, Texas, USA.
